# Facebook Recruitment Using Zip Codes to Improve Diversity in Health Research: Longitudinal Observational Study

**DOI:** 10.2196/17554

**Published:** 2020-06-05

**Authors:** Cornelia Pechmann, Connor Phillips, Douglas Calder, Judith J Prochaska

**Affiliations:** 1 Paul Merage School of Business University of California, Irvine Irvine, CA United States; 2 Stanford Prevention Research Center Department of Medicine Stanford University Stanford, CA United States

**Keywords:** smoking, advertisement, social media

## Abstract

**Background:**

Facebook’s advertising platform reaches most US households and has been used for health-related research recruitment. The platform allows for advertising segmentation by age, gender, and location; however, it does not explicitly allow for targeting by race or ethnicity to facilitate a diverse participant pool.

**Objective:**

This study looked at the efficacy of zip code targeting in Facebook advertising to reach blacks/African Americans and Hispanics/Latinos who smoke daily for a quit-smoking web-based social media study.

**Methods:**

We ran a general market campaign for 61 weeks using all continental US zip codes as a baseline. Concurrently, we ran 2 campaigns to reach black/African American and Hispanic-/Latino-identified adults, targeting zip codes ranked first by the percentage of households of the racial or ethnic group of interest and then by cigarette expenditure per household. We also ran a Spanish language campaign for 13 weeks, targeting all continental US zip codes but utilizing Facebook’s Spanish language targeting. The advertising images and language were common across campaigns. Costs were compared for advertisement clicks, queries, applications, and participants, and yields were compared for the final three outcomes. We examined outcomes before and after the Cambridge Analytica scandal that broke in March 2018. Finally, we examined 2 promoted Facebook features: lookalike audiences and audience network placement.

**Results:**

Zip code targeting campaigns were effective for yielding the racial or ethnic groups of interest. The black-/African American–focused versus general market campaign increased black/African American weekly queries (mean 9.48, SD 5.69 vs general market mean 2.83, SD 2.05; *P*<.001) and applicants (mean 1.11, SD 1.21 vs general market mean 0.54, SD 0.58; *P*<.001). The Hispanic-/Latino-focused versus general market campaign increased Hispanic/Latino weekly queries (mean 3.10, SD 2.16 vs general market mean 0.71, SD 0.48; *P*<.001) and applicants (mean 0.36, SD 0.55 vs general market mean 0.10, SD 0.14; *P*=.001). Cost metrics did not differ between campaigns at generating participants (overall *P*=.54). Costs increased post- versus prescandal for the black-/African American–focused campaign for queries (mean US $8.51, SD 3.08 vs US $5.87, SD 1.89; *P*=.001) and applicants (mean US $59.64, SD 35.63 vs US $38.96, SD 28.31; *P*=.004) and for the Hispanic-/Latino-focused campaign for queries (mean US $9.24, SD 4.74 vs US $7.04, SD 3.39; *P*=.005) and applicants (mean US $61.19, SD 40.08 vs US $38.19, SD 21.20; *P*=.001).

**Conclusions:**

Zip code targeting in Facebook advertising is an effective way to recruit diverse populations for health-based interventions. Audience network placement should be avoided. The Facebook lookalike audience may not be necessary for recruitment, with drawbacks including an unknown algorithm and unclear use of Facebook user data, and so public concerns around data privacy should be considered.

**Trial Registration:**

ClinicalTrial.gov NCT02823028; https://clinicaltrials.gov/ct2/show/NCT02823028

## Introduction

### Background

The distrust of health care and health-related studies among blacks/African Americans and Hispanics/Latinos has been documented extensively [1-3]. To combat racial and ethnic homogeneity in health-related research, emphasis must be placed on recruitment of diverse participants, especially in studies concerning diseases or products in which race or ethnicity is a factor [4,5]. Tobacco companies have traditionally advertised products to communities of color [6-8]. Their targeting methods have included placing advertisements on television shows, in print media, and on websites with high black/African American or Hispanic/Latino viewership [9,10]. Tobacco companies have also spatially targeted communities of color by placing billboards and bus advertisements in neighborhoods primarily comprising black/African American or Hispanic/Latino residents [11]. Tobacco companies have paid people to go into inner-city neighborhoods to hand out free samples of menthol (and sometimes regular) cigarettes in an effort to attract black/African American young adult and adult customers [12-14].

Facebook is a leading web-based social media platform for adults in the United States, with approximately 221 million monthly active users who represent about 69% of the adult population [15,16]. Facebook is also used at similar rates among whites, blacks/African Americans, and Hispanics/Latinos [17]. Facebook advertising has increasingly been used for health-related study recruitment [18]. Cost efficiency and widespread adult use, as well as detailed targeting features, make Facebook advertising a popular choice for study recruitment [18,19]. Participants recruited through the Facebook advertising platform were found to be demographically similar compared with traditional study recruitment methods, such as print materials [18,20]. In some cases, however, participant pools showed an overrepresentation of non-Hispanic white individuals [21]. Race and ethnicity are important aspects of study recruitment, and more emphasis is being placed on inclusion and reporting of racial or ethnic diversity in the participant pool, especially in health-related research [22]. However, there remains little discussion about how to target diverse racial or ethnic groups using Facebook advertising [23].

This paper discusses recruitment of racially and ethnically diverse participants by means of Facebook advertising for a quit-smoking web-based social media study. Our research team developed a web-based Twitter peer-support group intervention for quitting smoking called Tweet2Quit [24,25]. We recruited virtually all participants (N=980) using Facebook advertising and sought an ethnically diverse participant pool.

### Study Goals

This study evaluated 4 Facebook advertising campaigns that we used to recruit individuals for our quit-smoking web-based social media study. Our general market campaign reached all continental US zip codes, targeting individuals who expressed an interest in smoking or quitting smoking on Facebook, and it served as our baseline. Two additional Facebook campaigns targeted primarily black/African American or Hispanic/Latino zip codes with high cigarette expenditures. Our final Facebook campaign targeted Spanish language speakers living in continental US zip codes who expressed an interest in smoking or quitting smoking on Facebook. For each campaign, we examined yields and costs for 4 standard advertising campaign outcomes: advertisement clicks, queries, applications, and participants. We hypothesized that zip code campaigns would generate higher yields of diverse ethnic groups without higher costs.

In addition, we compared costs before and after major news broke in March 2018 concerning the Facebook privacy scandal involving Cambridge Analytica’s alleged breach of privacy of 50 million Facebook users, which raised serious concerns about the protection of private user information. Finally, we examined the costs associated with 2 features Facebook recommends to its advertisers, use of lookalike audiences and audience network placement, and compared these to our general market campaign.

## Methods

### Facebook Advertising Campaigns

Facebook does not allow for direct demographic targeting of advertising by users’ ethnicity or race. The main demographics that Facebook advertisers can choose are age and gender. These demographics are offered because when Facebook users create their accounts, they are required to specify their age and gender. Advertisers can further narrow their audience by using the 3 sections labeled *Demographics*, *Interests*, and *Behaviors*. The *Demographics* and *Interests* sections do not include race or ethnicity. The *Behaviors* section includes Multicultural Affinity, which allows advertisers to choose African American, Asian American, or Hispanic affinity. Facebook does not provide information on how individuals are classified into these behavioral affinity categories or what data are used. In addition, categories within the *Interests* and *Behaviors* sections change often, which then requires advertisers to recreate existing advertising campaigns using new options. Furthermore, advertising using Multicultural Affinity is restricted by Facebook and can lead to extended advertising review times and automated advertisement disapproval [26]. Due to these reasons, we used more standard zip code targeting in our study.

As a baseline, we created a general market campaign, selecting all continental US zip codes. The advertising images and language were common in all 4 advertising campaigns we studied. In addition, in all 4 campaigns, we restricted our advertisements to be seen by individuals aged between 21-59 years. We required individuals to be ≥21 years because many states restrict the sale of tobacco to these individuals. We set the maximum age at 59 to focus on younger and middle-aged adults rather than retirees. Moreover, in all 4 of our advertising campaigns, we targeted appropriate study participants based on their Facebook interests. We showed our advertisements only to individuals who expressed an interest in smoking-related topics such as quitting smoking, nicotine, and tobacco or cigarettes, while logged into Facebook.

For our black-/African American–focused and Hispanic-/Latino-focused campaigns, we also used more specific zip code targeting. We obtained data on all continental US zip codes and sorted the zip codes from highest to lowest based on the percentage of households in the target racial or ethnic group to ensure that those racial or ethnic groups were reached. After this, we sorted the zip codes from highest to lowest based on the mean annual household expenditure on cigarettes to try to ensure that we reached smokers. We removed any zip codes with fewer than 100 households. We then selected the top 1000 zip codes because Facebook only allowed us to upload 1000 customized zip codes out of the 41,702 total zip codes in the United States [27-29]. All chosen zip codes had high percentages of ethnic households and high cigarette expenditures. The 1000 zip codes we used for the black-/African American–focused campaign ranged from 53% to 99% in terms of the households in this ethnic group (mean 70%, SD 0.13%), with annual cigarette expenditures from US $154 to US $568 (mean US $329, SD 62.49) per household. The 1000 zip codes we used for the Hispanic-/Latino-focused campaign ranged from 53% to 99% (mean 71%, SD 0.13%) in terms of the households in this ethnic group, with annual cigarette expenditures from US $70 to US $506 (mean US $266, SD 69.86) per household. We uploaded the targeted zip codes for each campaign on Facebook.

Finally, for our Spanish language campaign, we used all continental US zip codes; however, we selected Spanish as the spoken language, using Facebook’s designated Language Targeting feature. This feature allows advertisers to show advertisements to users who speak a specific language and is not related to targeting based on the *Behaviors* or *Interests* sections. Spanish language targeting was chosen to increase the number of Hispanic/Latino applicants to our program. Advertisements across all campaigns were identical, as noted above. Advertising imagery and wording were chosen using Facebook A/B testing campaigns placed in all US zip codes. A/B testing allows advertisers to run nearly identical, simultaneous campaigns to test a singular variable, such as an advertising image.

Data on the ethnic makeup of US zip codes came from the US Census Bureau’s American Community Survey and are publicly available [27]. Data on cigarette expenditures came from the US Bureau of Labor Statistics’ Consumer Expenditure Survey and, although not publicly available at the zip code level, can be purchased from Experian [28,30].

### Outcome Measures

Our study compared 4 different Facebook advertising campaigns: A general market campaign, a black-/African American–focused zip code campaign, a Hispanic-/Latino-focused zip code campaign, and a Spanish language campaign. We compared these campaigns on 4 standard outcome measures: advertisement clicks, queries, applicants, and participants. Our advertisement click measure showed whether an individual tapped or clicked on our web-based advertisement, which automatically directed the individual to our study website. Hence, our advertisement click measure assessed if a campaign possibly brought interested individuals to our website. On our website, individuals could read about the study and fill out a short query form that asked for their contact information (name and email) and their race or ethnicity. Our query measure assessed whether the campaign resulted in individuals providing contact information. Individuals who provided contact information received our full screening survey, and if they finished the survey, they were considered applicants. Hence, our applicant measure assessed whether the campaign resulted in individuals completing the study screener, regardless of whether they passed or failed. Finally, our participant measure assessed whether the campaign resulted in individuals getting enrolled into the study.

Our initial Facebook advertising testing began in October 2016 and included testing of the Facebook-recommended audience network placement. We began recruitment for our study in January 2017 but did not commence our sophisticated Pixel-based measurement of our Facebook campaigns (explained below) until mid-June 2017 and that continued until September 2018 for a total of 61 weeks. The Facebook scandal occurred in March 2018 (Multimedia Appendix 1).

### Descriptive Statistics on Overall Campaign Response

Across our 4 Facebook campaigns, we received a total of 92,677 advertisement clicks between June 20, 2017, and September 9, 2018. The general market campaign received 66,681 advertisement clicks, the black-/African American–focused campaign received 12,544 advertisement clicks, and the Hispanic-/Latino-focused campaign received 10,969 advertisement clicks. Our shorter Spanish language campaign received 2483 advertisement clicks between June 20, 2017, and September 30, 2017. On average, about 13.92% (12,898/92,677) of individuals who clicked our advertisement filled out the interest form, constituting a query. After receiving an email with a link to our web-based screening survey, about 19.31% (2490/12,898) of individuals completed the survey and about 20.32% (506/2490) of these applicants were enrolled in our study. We could not capture ethnicity at the advertisement click stage because we had no mechanism for doing this. However, we began collecting self-reported race or ethnicity at the query stage and continued this through the applicant and participant stages.

We examined whether study exclusion was related to the individual’s race or ethnicity. Race or ethnicity related to study exclusion due to a health contraindication (eg, pregnancy), in that blacks/African Americans (69/259, 26.6%) were more likely to be excluded for health reasons than non-Hispanic whites (295/1544, 19.11%; *Χ^2^*_1_=7.8; *P*=.005). Ethnicity related to study exclusion because of refusing mobile phone verification, in that whites (308/1544, 19.95%) were more likely to be excluded for this reason than blacks/African Americans (36/259, 13.9%; *Χ^2^*_1_=5.3; *P*=.02). Ethnicity related to study exclusion because of smoking too few cigarettes per day to permit use of the study-provided nicotine replacement therapy, in that blacks/African Americans (11/259, 4.2%) were more likely to be excluded for this reason than whites (28/1544, 1.81%; *Χ^2^*_1_=6.2; *P*=.01). No other effects were significant (all *P>*.17; Multimedia Appendix 2).

## Results

### Results Regarding Yields by Racial or Ethnic Group

#### Overview of Yield Analysis

Facebook’s basic advertisement reporting system measures advertisement clicks by default. To obtain the query, applicant, and participant metrics, we utilized a Facebook Pixel, which is a web code our website developer installed to measure and track actions Facebook users performed on our study website. The Facebook Pixel also tracked which of our Facebook campaigns drew them to our website [31]. We installed the Facebook Pixel in mid-June 2017. Our yield analysis is based on weekly data from June 20, 2017, to September 30, 2018, excluding major holidays (n=61 weeks), except Spanish language data that are from June 20, 2017, to September 30, 2017 (n=15 weeks). Although the Facebook Ads Manager provides daily yields, we aggregated the daily data to weekly data for accuracy and smoothing (eg, staff did not work weekends to convert queries to applicants). For all statistical analyses, we used analysis of variance, after which we conducted two-tailed pairwise *t* tests that compared specific campaigns (eg, general market vs Spanish language) using the Sidak correction for multiple comparisons.

We could not directly compare campaign yields because expenditures differed by campaign (eg, our budget for the general market campaign was considerably higher than for the black-/African American–focused and Hispanic-/Latino-focused campaigns). To compare yields across campaigns, we standardized expenditures to US $140 per week (US $20 per day), reflecting our average budget for the 2 main ethnic campaigns. For example, on a given week, we may have spent US $700 on the general market campaign versus US $140 on the black-/African American–focused campaign. If we received 100 queries from the general market campaign, we divided this number by 5 and estimated the yield as 20 based on a standardized expenditure of US $140 per week (US $700/5=US $140). This standardized yield of 20 for the general market campaign would be compared with the yield of the black-/African American–focused campaign that ran at US $140.

#### Non-Hispanic White Query Yields

**Figure 1:**
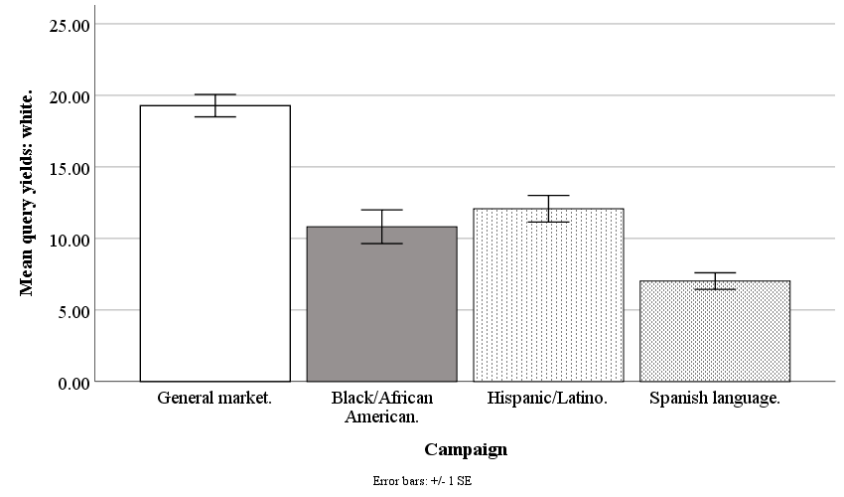
Mean query yields of whites for Facebook advertising campaigns.

The Facebook campaigns differed on weekly mean counts of queries by non-Hispanic whites (*F*_3,194_=19.79; *P*<.001), with the general market–focused campaign at 19.28 people (SD 6.11), black-/African American–focused at 10.82 people (SD 9.20), Hispanic-/Latino-focused at 12.10 people (SD 7.25), and Spanish language–focused at 7.02 people (SD 2.24). The query yield of whites was higher for the general market campaign than for the black-/African American–focused (*t*_1,194_=6.33; *P*<.001), Hispanic-/Latino-focused (*t*_1,194_=5.39; *P*<.001), or Spanish language–focused campaigns (*t*_1,194_=5.77; *P*<.001). The query yield of whites was similar for the black-/African American–focused campaign versus Hispanic-/Latino-focused (*t*_1,194_=0.94; *P*=.92) or Spanish language campaigns (*t*_1,194_=1.79; *P*=.37). The weekly mean count of whites was similar for the Hispanic-/Latino-focused versus Spanish language campaign (*t*_1,194_=2.38; *P*=.11; Figure 1).

#### Black/African American Query Yield

**Figure 2:**
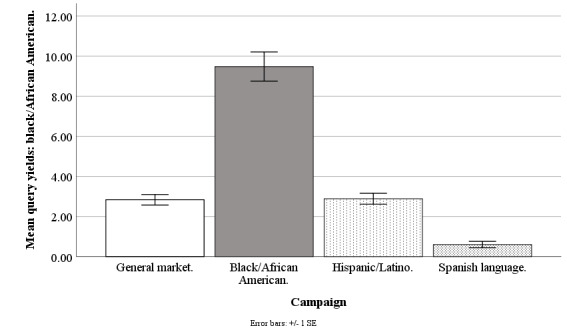
Mean query yields of blacks/African Americans for Facebook advertising campaigns.

The Facebook campaigns differed on weekly mean counts of queries by blacks/African Americans (*F*_3,194_=53.96; *P*<.001), with the general market–focused campaign at 2.83 people (SD 2.05), black-/African American–focused at 9.48 people (SD 5.69), Hispanic-/Latino-focused at 2.89 people (SD 2.12), and Spanish language–focused at 0.60 people (SD 0.62). The query yield of blacks/African Americans was higher for the black-/African American–focused campaign than for the general market–focused (*t*_1,194_=10.29; *P*<.001), Hispanic-/Latino-focused (*t*_1,194_=10.21; *P*<.001), or Spanish language–focused campaigns (*t*_1,194_=8.64; *P*<.001). The query yield of blacks/African Americans was similar for the general market–focused versus Hispanic-/Latino-focused (*t*_1,194_=0.08; *P*=.99) or Spanish language–focused campaigns (*t*_1,194_=2.17; *P*=.17). The query yield of blacks/African Americans was similar for the Hispanic-/Latino-focused versus Spanish language–focused campaign (*t*_1,194_=2.23; *P*=.15; Figure 2).

#### Hispanic/Latino Query Yield

**Figure 3:**
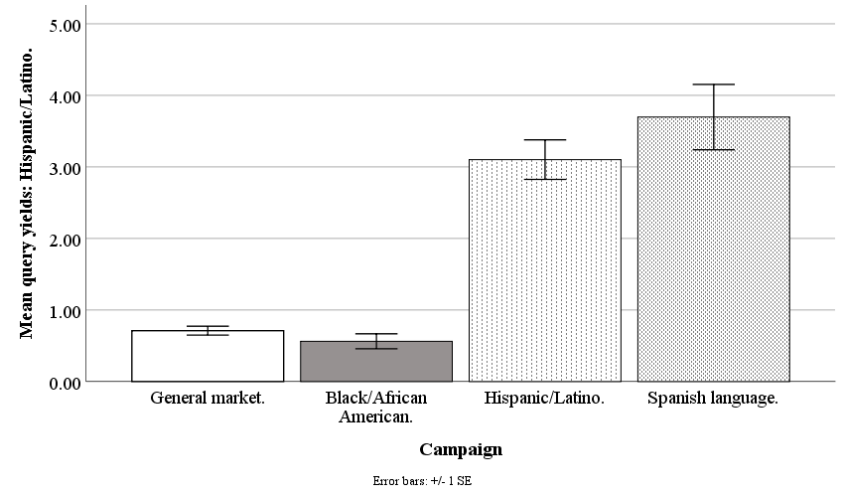
Mean query yields of Hispanics/Latinos for Facebook advertising campaigns.

The Facebook campaigns differed on weekly mean counts of queries by Hispanics/Latinos (*F*_3,194_=54.29; *P*<.001), with the general market–focused campaign at 0.71 people (SD 0.48), black-/African American–focused at 0.56 people (SD 0.82), Hispanic-/Latino-focused at 3.10 people (SD 2.16), and Spanish language–focused at 3.70 people (SD 1.77). The query yield of Hispanics/Latinos was higher for the Hispanic-/Latino-focused campaign than for the general market–focused (*t*_1,194_=9.45; *P*<.001) or black-/African American–focused campaigns (*t*_1,194_=10.04; *P*<.001) but was similar to the Spanish language–focused campaign (*t*_1,194_=1.48; *P*=.60). The query yield of Hispanics/Latinos was similar for the general market–focused campaign as compared with the black-/African American–focused campaign (*t*_1,194_=0.58; *P*=.99) but was lower than that of the Spanish language–focused campaign (*t*_1,194_=7.43; *P*<.001). The query yield of Hispanics/Latinos was higher for the Spanish language–focused versus African American–focused campaign (*t*_1,194_=7.79; *P*<.001; Figure 3).

#### Non-Hispanic White Applicant Yield

**Figure 4:**
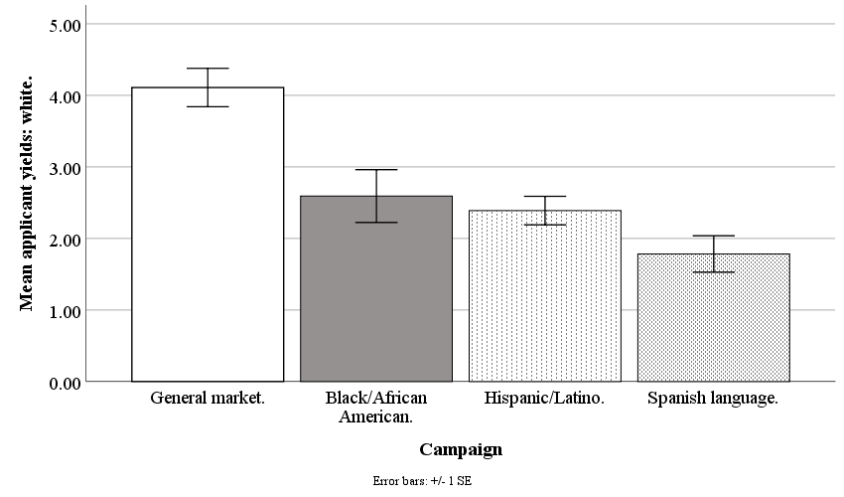
Mean applicant yields of whites for Facebook advertising campaigns.

The Facebook campaigns differed on weekly mean counts of applicants who were non-Hispanic white (*F*_3,190_=9.00; *P*<.001), with the general market–focused campaign at 4.12 (SD 2.08), black-/African American–focused at 2.59 (SD 2.86), Hispanic-/Latino-focused at 2.39 (SD 1.55), and Spanish language–focused at 1.78 (SD 0.95). The applicant yield of whites was higher for the general market–focused campaign than for the black-/African American–focused (*t*_1,190_=3.84; *P*=.001), Hispanic-/Latino-focused (*t*_1,190_=4.35; *P*<.001), or Spanish language–focused campaigns (*t*_1,190_=3.62; *P*=.002). The applicant yield of whites was similar for the black-/African American–focused versus Hispanic-/Latino-focused (*t*_1,190_=0.51; *P*=.99) or Spanish language–focused campaigns (*t*_1,190_=1.26; *P*=.76). The applicant yield of whites was similar for the Hispanic-/Latino-focused versus Spanish language–focused campaign (*t*_1,190_=0.95; *P*=.92; Figure 4).

#### Black/African American Applicant Yields

**Figure 5:**
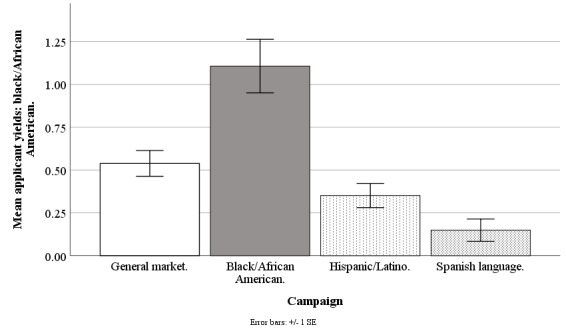
Mean applicant yields of blacks/African Americans for Facebook advertising campaigns.

The Facebook campaigns differed on weekly mean counts of applicants who were black/African American (*F*_3,190_=11.19; *P*<.001), with the general market–focused campaign at 0.54 (SD 0.58), black-/African American–focused at 1.11 (SD 1.21), Hispanic-/Latino-focused at 0.35 (SD 0.55), and Spanish language–focused at 0.15 (SD 0.24). The applicant yield of blacks/African Americans was higher for the black-/African American–focused campaign compared with the general market–focused (*t*_1,190_=3.84; *P*=.001), Hispanic-/Latino-focused (*t*_1,190_=5.11; *P*<.001), or Spanish language–focused campaigns (*t*_1,190_=3.96; *P*=.001). The applicant yield of blacks/African Americans was similar for the general market–focused versus Hispanic-/Latino-focused (*t*_1,190_=1.27; *P*=.75) or Spanish language–focused campaigns (*t*_1,190_=1.62; *P*=.49). The applicant yield of blacks/African Americans was similar for the Hispanic-/Latino-focused versus Spanish language–focused campaign (*t*_1,190_=0.84; *P*=.95; Figure 5).

#### Hispanic/Latino Applicant Yield

**Figure 6:**
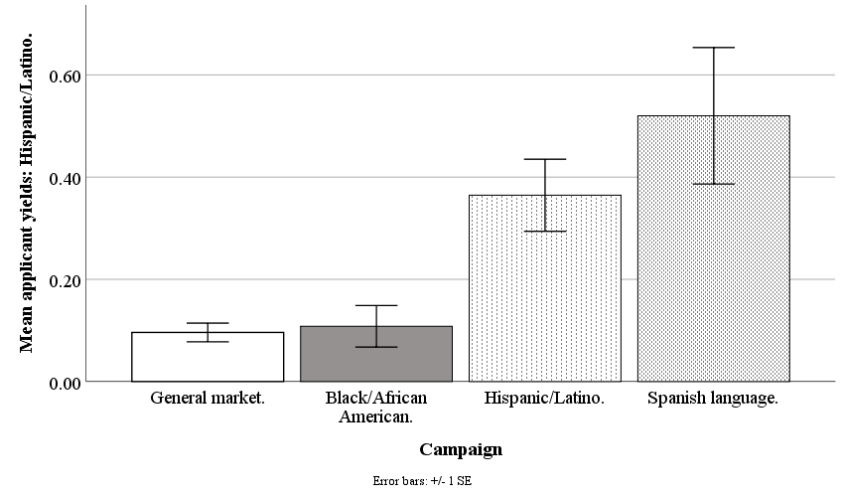
Mean applicant yields of Hispanics/Latinos for Facebook advertising campaigns.

The Facebook campaigns differed on weekly mean counts of applicants who were Hispanic/Latino (*F*_3,190_=9.48; *P*<.001), with the general market–focused campaign at 0.10 (SD 0.14), black-/African American–focused at 0.11 (SD 0.32), Hispanic-/Latino-focused at 0.36 (SD 0.55), and Spanish language–focused at 0.52 (SD 0.50). The applicant yield of Hispanics/Latinos was higher for the Hispanic-/Latino-focused campaign compared with general market–focused (*t*_1,190_=3.83; *P*=.001) or black-/African American–focused campaigns (*t*_1,190_=3.66; *P*=.002) but similar to that of Spanish language–focused campaign (*t*_1,190_=1.37; *P*=.68). The applicant yield of Hispanics/Latinos was similar for the general market–focused campaign compared with the black-/African American–focused campaign (*t*_1,190_=0.17; *P*=.99) but lower than that of the Spanish language–focused campaign (*t*_1,190_=3.72; *P*=.002). The applicant yield of Hispanics/Latinos was higher for the Spanish language–focused campaign versus the black-/African American-focused campaign (*t*_1,190_=3.61; *P*=.002; Figure 6).

#### Non-Hispanic White Participant Yields

**Figure 7:**
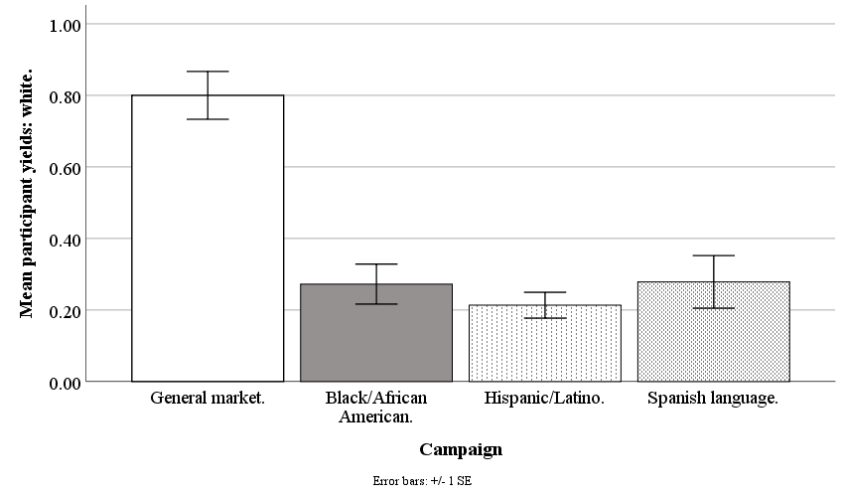
Mean participant yields of whites for Facebook advertising campaigns.

The Facebook campaigns differed on weekly mean counts of participants who were non-Hispanic white (*F*_3,190_=24.96; *P*<.001), with the general market–focused campaign at 0.80 (SD 0.52), black-/African American–focused at 0.27 (SD 0.43), Hispanic-/Latino-focused at 0.21 (SD 0.28), and Spanish language–focused at 0.28 (SD 0.28). The participant yield of whites was higher for the general market–focused campaign than for the black-/African American–focused (*t*_1,190_=6.95; *P*<.001), Hispanic-/Latino-focused (*t*_1,190_=7.72; *P*<.001), or Spanish language–focused campaigns (*t*_1,190_=4.24; *P*<.001). The participant yield of whites otherwise did not differ across campaigns (all *P>*.97; Figure 7).

#### Black/African American Participant Yields

**Figure 8:**
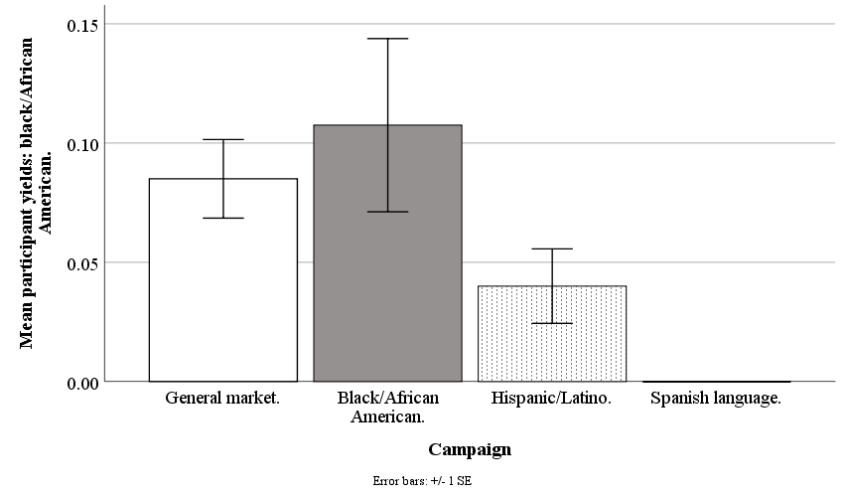
Mean participant yields of blacks/African Americans for Facebook advertising campaigns.

The Facebook campaigns marginally differed on weekly mean counts of participants who were black/African American (*F*_3,190_=2.14; *P*=.10), with the general market–focused campaign at 0.09 (SD 0.13), black/African American–focused at 0.11 (SD 0.28), Hispanic-/Latino-focused at 0.04 (SD 0.12), and Spanish language–focused at .001 (SD 0.001). However, none of the pairwise comparisons between specific campaigns were significant (all *P*>.25; Figure 8).

#### Hispanic/Latino Participant Yields

**Figure 9:**
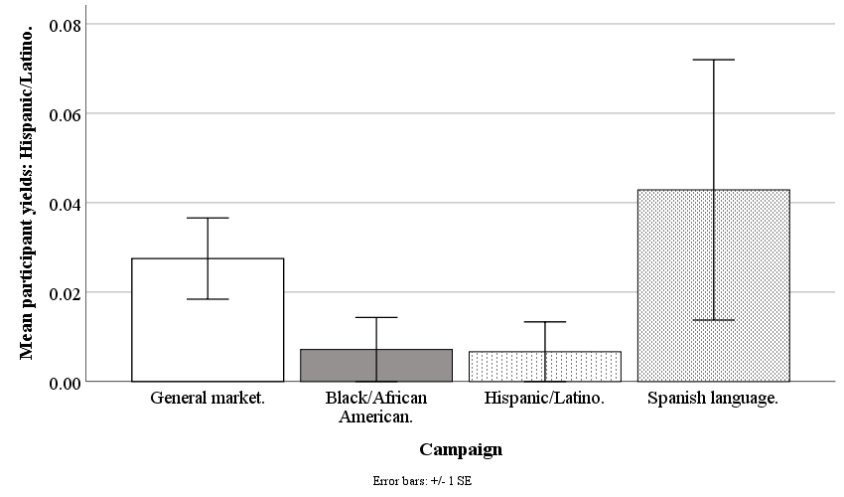
Mean participant yields of Hispanics/Latinos for Facebook advertising campaigns.

The Facebook campaigns marginally differed on the weekly mean counts of participants who were Hispanic/Latino (*F*_3,190_=2.25; *P*=.08), with the general market–focused campaign at 0.03 (SD 0.07), black-/African American–focused at 0.01 (SD 0.06), Hispanic-/Latino-focused at 0.01 (SD 0.05), and Spanish language–focused at 0.04 (SD 0.11). However, none of the pairwise comparisons between specific campaigns were significant (all *P*>.31; Figure 9).

### Results Regarding Costs by Ethnic Group

#### Overview of Cost Analysis

**Figure 10:**
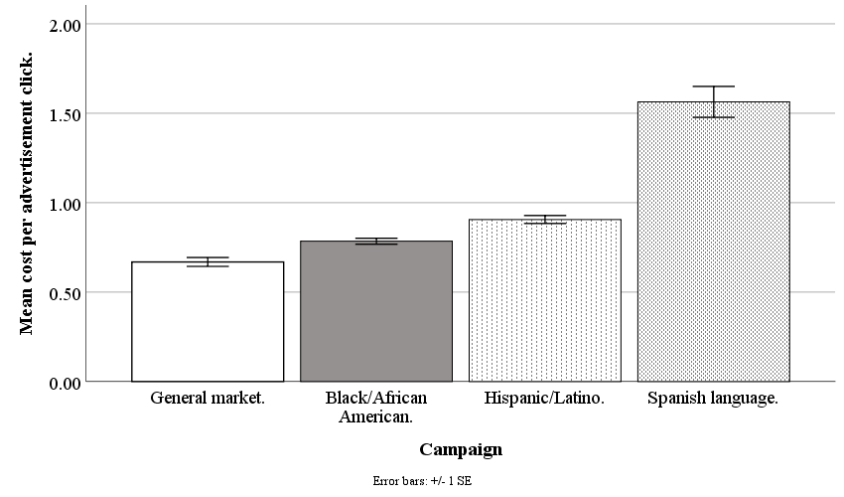
Mean costs per advertisement click for Facebook advertising campaigns.

**Figure 11:**
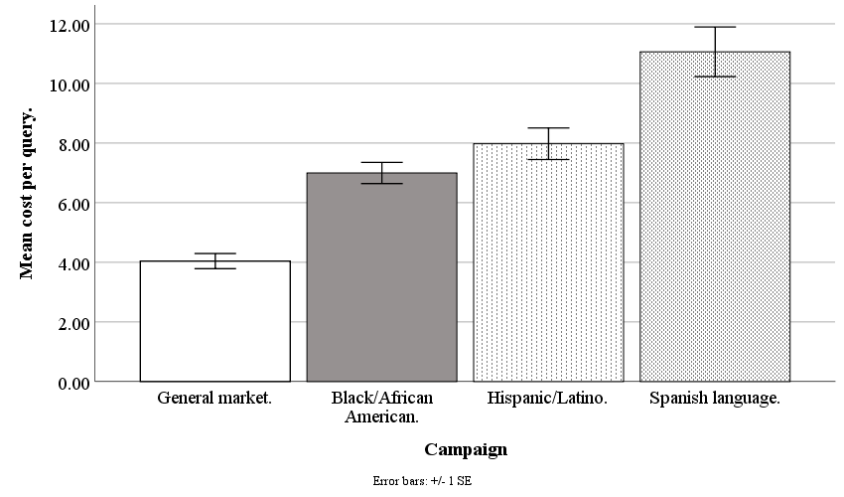
Mean costs per query for Facebook advertising campaigns.

**Figure 12:**
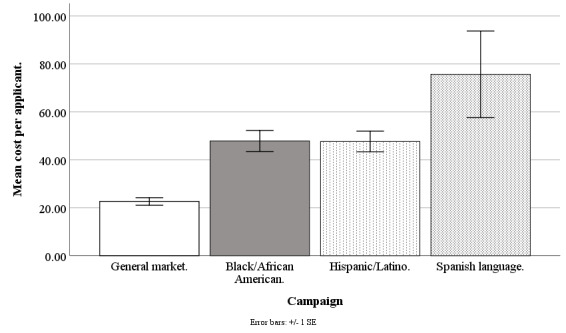
Mean costs per applicant for Facebook advertising campaigns.

**Figure 13:**
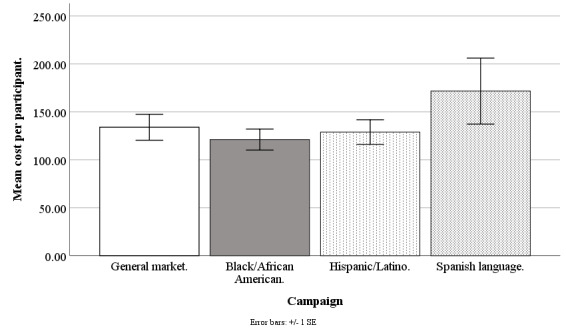
Mean costs per participant for Facebook advertising campaigns.

Facebook advertising costs are available at the campaign level (for instance, for a Hispanic-/Latino-focused campaign) but not at the individual level (eg, for a Hispanic/Latino individual) because at the individual level, internal auctions are used to determine if an advertisement is shown, and Facebook does not share these individual-level costs with its advertisers [32]. The Facebook Ads Manager only provides costs per campaign and per day, and so our best estimate of the recruitment cost of any individual regardless of ethnicity was the average cost for the campaign on the day in which the recruitment took place. Daily data were aggregated to weekly data for accuracy and smoothing (eg, staff did not work weekends to convert queries to applicants). Our cost analysis is based on weekly data from June 20, 2017, to September 17, 2018, excluding major holidays (n=61 weeks), except the Spanish language data that are from June 20, 2017, to September 30, 2018 (n=15 weeks). Refer to Figures 10-13 for campaign costs by ethnicity at the advertisement click, query, applicant, and participant stages.

#### Cost per Advertisement Click

The Facebook campaigns differed on cost per advertisement click (*F*_3,194_=97.18; *P*<.001), with the general market–focused campaign at US $0.67 (SD 0.19), black-/African American–focused at US $0.78 (SD 0.13), Hispanic-/Latino-focused at US $0.91 (SD 0.17), and Spanish language-focused at US $1.56 (SD 0.34). The cost per advertisement click was lower for the general market–focused campaign, compared with black-/African American–focused (*t*_1,194_=3.41; *P*=.004), Hispanic-/Latino-focused (*t*_1,194_=6.97; *P*<.001), or Spanish language–focused (*t*_1,194_=16.57; *P*<.001). The cost per advertisement click was lower for the black-/African American–focused campaign than for the Hispanic-/Latino-focused (*t*_1,194_=3.56; *P*=.002) or Spanish language-focused (*t*_1,194_=14.43; *P*<.001) campaigns, and lower for the Hispanic-/Latino-focused than for the Spanish language–focused campaign (*t*_1,194_=12.49; *P*<.001; Figure 10).

#### Cost per Query

The Facebook campaigns differed in cost per query (*F*_3,194_=28.42; *P*<.001), with the general market–focused campaign at US $4.04 (SD 1.99), black-/African American–focused at US $7.00 (SD 2.77), Hispanic-/Latino-focused at US $7.98 (SD 4.13), and Spanish language–focused at US $11.06 (SD 3.23). The cost per query was lower for the general market–focused campaign than for the black-/African American–focused (*t*_1,194_=5.26; *P*<.001), Hispanic-/Latino-focused (*t*_1,194_=7.01; *P*<.001), or Spanish language–focused campaigns (*t*_1,194_=7.84; *P*<.001). The cost per query was lower for the black-/African American–focused campaign than for the Hispanic-/Latino-focused (*t*_1,194_=3.56; *P*=.002) or Spanish language–focused campaigns (*t*_1,194_=4.54; *P*<.001), and lower for the Hispanic-/Latino-focused than for the Spanish language–focused campaign (*t*_1,194_=3.45; *P*=.004; Figure 11).

#### Cost per Applicant

The Facebook campaigns differed in cost per applicant (*F*_3,182_=13.80; *P*<.001), with the general market–focused at US $22.61 (SD 12.21), black-/African American–focused at US $47.82 (SD 33.00), Hispanic-/Latino-focused at US $47.63 (SD 32.16), and Spanish language–focused at US $75.65 (SD 67.60). The cost per applicant was lower for the general market–focused campaign, compared with the black-/African American–focused (*t*_1,182_=4.26; *P*<.001), Hispanic-/Latino-focused (*t*_1,182_=4.23; *P*<.001), or Spanish language–focused campaigns (*t*_1,182_=5.61; *P*<.001). The cost per applicant was comparable for the black-/African American–focused campaign than for the Hispanic-/Latino-focused campaign (*t*_1,182_=0.03; *P*=.99) but lower than that of the Spanish language–focused campaign (*t*_1,182_=2.92; *P*=.02). The cost per applicant was also lower for the Hispanic-/Latino-focused than for the Spanish language–focused campaign (*t*_1,182_=2.94; *P*=.02; Figure 12).

#### Cost per Participant

The Facebook campaigns were comparable on cost per participant (*F*_3,121_=0.73; *P*=.54), with the general market–focused at US $133.90 (SD 103.92), black-/African American–focused at US $121.05 (SD 60.02), Hispanic-/Latino-focused at US $128.90 (SD 67.86), and Spanish language–focused at US $171.66 (SD 97.37). Likewise, comparing the specific campaigns pairwise, there were no differences in cost per participant (all *P*>.62; Figure 13).

### Results of Cambridge Analytica Scandal

#### Overview of Analysis of Scandal Effects

**Figure 14:**
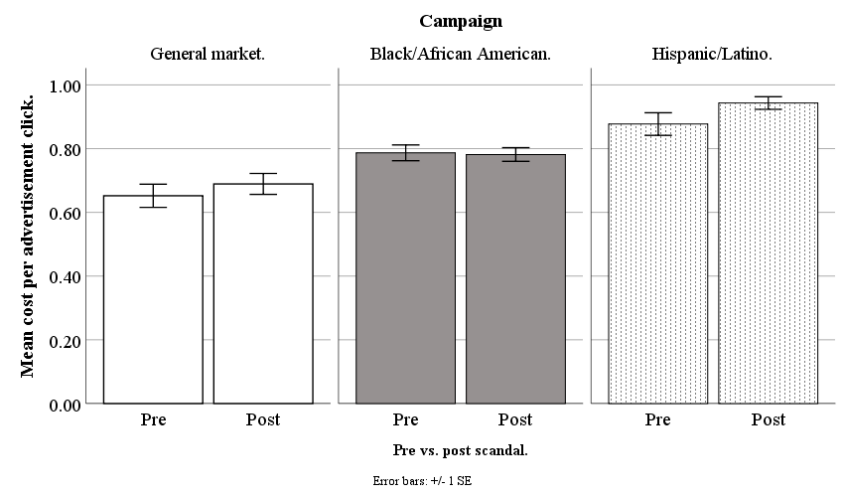
Mean costs per advertisement click for Facebook advertising campaigns before versus after privacy scandal.

**Figure 15:**
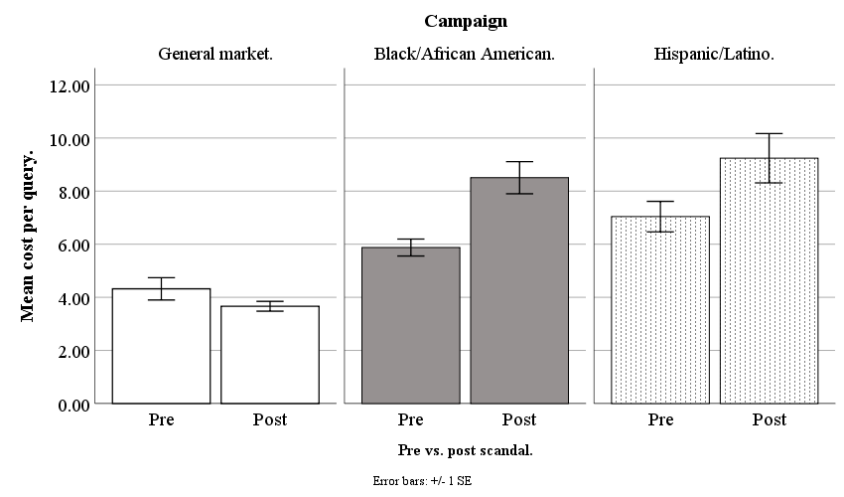
Mean costs per query for Facebook advertising campaigns before versus after privacy scandal.

**Figure 16:**
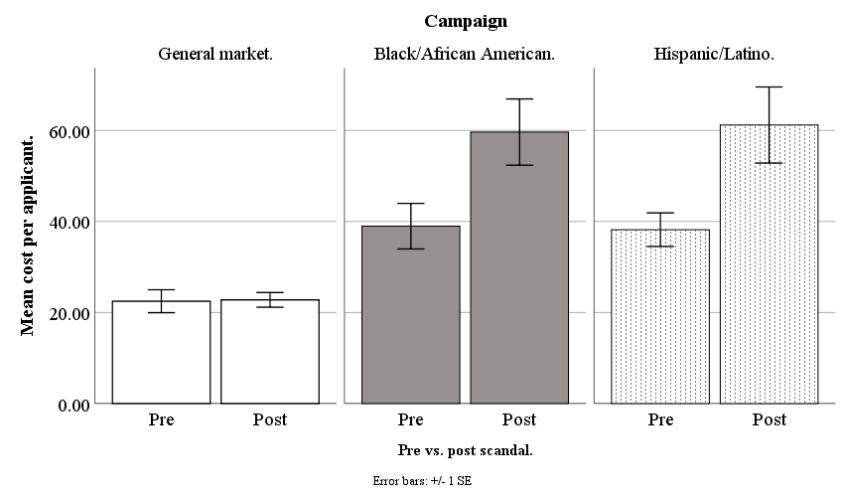
Mean costs per applicant for Facebook advertising campaigns before versus after privacy scandal.

**Figure 17:**
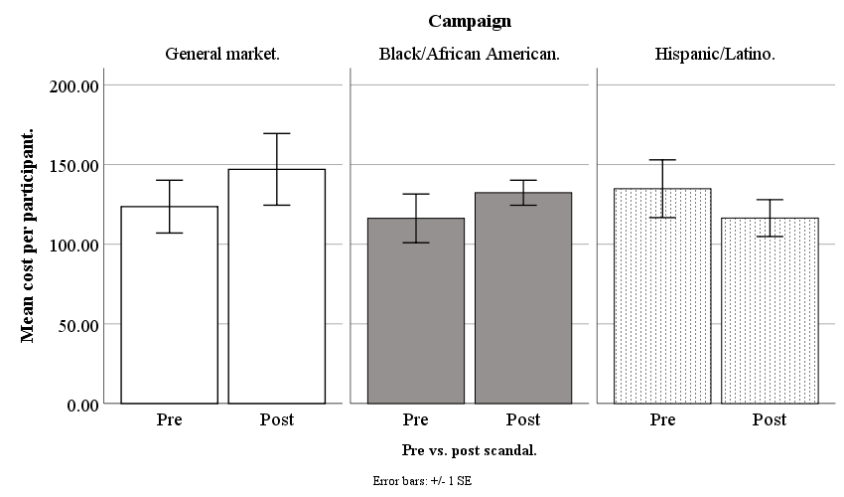
Mean costs per participant for Facebook advertising campaigns before versus after privacy scandal.

In March 2018, Facebook started to receive negative publicity because a third-party company, Cambridge Analytica, had allegedly harvested information from more than 50 million Facebook accounts without users’ permission [33]. Our advertisements ran before and after this scandal broke, and so we compared our costs for advertisement clicks, queries, applicants, and participants across these 2 time periods, to determine whether the Facebook privacy scandal increased our recruitment costs, and whether this depended on the campaign: general market–focused, black-/African American–focused, or Hispanic-/Latino-focused (our Spanish language campaign was no longer running). Our scandal analysis was based on weekly data from June 20, 2017, to September 17, 2018, excluding major holidays (n=61 weeks). Because the original New York Times article on the scandal appeared on March 17, 2018, all weeks before March 20, 2018, (n=33) were treated as prescandal and the remaining weeks (n=28) were treated as postscandal. Refer to Figures 14-17 for our costs by campaign pre- versus postscandal.

#### Cost per Advertisement Click

On cost per advertisement click, the effect for pre- versus postscandal was not significant (*F*_1,177_=1.67; *P*=.20). Additionally, there were no significant differences in cost per advertisement click when we conducted specific pairwise comparisons of the campaigns (all *P*>.13; Figure 14).

#### Cost per Query

On cost per query, there was a significant effect for pre- versus postscandal (*F*_1,177_=9.93; *P*=.002) and a scandal by campaign type 2-way interaction (*F*_1,177_=5.46; *P*=.005), meaning the scandal effect varied by campaign. For the general market–focused campaign, there was no scandal effect on cost per query (*F*_1,177_=0.74; *P*=.39), with prescandal at US $4.32 (SD 2.49) and postscandal at US $3.66 (SD 0.94). For the black-/African American–focused campaign, there was a scandal effect on cost per query (*F*_1,177_=11.84; *P*=.001), with prescandal at US $5.87 (SD 1.89) and postscandal higher at US $8.51 (SD 3.08). Likewise, for the Hispanic-/Latino-focused campaign, there was a scandal effect on cost per query (*F*_1,177_=8.28; *P*=.01), with prescandal at US $7.04 (SD 3.39) and postscandal higher at US $9.24 (SD 4.74; Figure 15).

#### Cost per Applicant

On cost per applicant, there was a significant effect for pre- versus postscandal (*F*_1,166_=13.29; *P*<.001) and a scandal by campaign type 2-way interaction (*F*_1,166_=3.30; *P*=.04), meaning the scandal effect varied by campaign. For the general market–focused campaign, there was no scandal effect on cost per applicant (*F*_1,166_=.002; *P*=.97), with prescandal at US $22.48 (SD 14.68) and postscandal at US $22.77 (SD 8.19). For the black-/African American–focused campaign, there was a scandal effect on cost per applicant (*F*_1,166_=8.65; *P*=.004), with prescandal at US $38.96 (SD 28.31) and postscandal higher at US $59.64 (SD 35.63). Similarly, for the Hispanic-/Latino-focused campaign, there was a scandal effect on cost per applicant (*F*_1,166_=10.57; *P*=.001), with prescandal at US $38.19 (SD 21.20) and postscandal higher at US $61.19 (SD 40.08; Figure 16).

#### Cost per Participant

On cost per participant, the effect for pre- versus postscandal was not significant (*F*_1,111_=0.15; *P*=.70). Additionally, there were no significant differences in cost per participant when we conducted specific pairwise comparisons of the campaigns (all *P*>.31; Figure 17).

### Results Regarding Lookalike Audience

We used Facebook’s lookalike audience feature for a short time to determine if it reduced our recruitment costs relative to our baseline general market–focused campaign. Facebook’s lookalike audience feature involves a proprietary method of targeting individuals similar to a chosen source audience, that is, an audience that responded favorably to a past campaign based on a specific website response as recorded by the Facebook Pixel. On the basis of the designated source audience, Facebook seeks to generate a similarly interested audience [34]. We designated our source audience as individuals who filled out our query form on our study website, which was recorded by our website’s Facebook Pixel. Our analysis was based on daily data from January 3, 2018, to March 26, 2018 (n=26 days). It compared the Facebook lookalike campaign that sought to optimize our queries to our standard general market–focused campaign, which ran simultaneously. We assessed cost per advertisement click, query, and applicant; however, cost per participant could not be estimated due to the short time window. This test was ended soon after it began because of privacy concerns. Facebook’s lookalike audience feature has the drawbacks of an unknown and unknowable algorithm and unclear use of Facebook user data to create the algorithm, which might possibly involve using Facebook users’ network of friends. Therefore, we could not fully describe the data collection approach to our potential study participants and ask for their informed consent or obtain their friends’ consent, if needed.

The cost per advertisement click for the lookalike audience campaign was lower than the general market–focused campaign (*F*_1,50_=26.64; *P*<.001), with the lookalike audience at US $0.73 (SD 0.30) and general market–focused at US $1.07 (SD 0.14). However, the cost per query for the lookalike audience campaign did not differ from the general market campaign (*F*_1,49_=0.23; *P*=.64), with lookalike audience at US $3.98 (SD 2.24) and general market at US $4.27 (SD 2.19). The cost per applicant for the lookalike audience campaign also did not differ from the general market campaign (*F*_1,40_=2.75; *P*=.11), with lookalike audience at US $15.58 (SD 6.07) and general market at US $20.13 (SD 10.13).

### Results Regarding Audience Network Placement

Audience network placement is a feature recommended by Facebook to its advertisers, which involves placing their advertisements on other websites or mobile apps rather than on Facebook itself, to reach a broader audience. We assessed whether using Facebook’s audience network placement reduced recruitment costs. We tried out the audience network placement very early on, before we began study recruitment, and so only costs per advertisement click and query were available. Facebook automatically provides advertisement click data, and we were able to record queries because we had installed and monitored our query form on our website. We used the audience network placement on its own during 2 test weeks in 2016, so we could attribute all queries to it. Later on, our Facebook Pixel would identify queries by campaign.

Here, we compare data from our general market campaign that used audience network placement (August 3, 2016-August 10, 2016, and August 23, 2016-August 30, 2016, n=16 days) to standard advertisement placement on Facebook alone (1 year later, on exactly the same dates, n=16 days). The analysis shows that the cost per advertisement click was lower when the audience network placement was used compared with placement on Facebook alone (*F*_1,30_=262.10; *P*<.001), with audience network at US $0.11 (SD 0.05) and Facebook alone at US $0.69 (SD 0.13). However, the cost per query was substantially higher when audience network placement was used compared with placement on Facebook alone (*F*_1,30_=4.26; *P*=.048), with the audience network at US $13.40 (SD 14.83) and Facebook alone at US $5.62 (SD 2.76). After 2 test weeks, based on noticeably poor query results, we stopped using the audience network placement.

## Discussion

### Principal Findings on Zip Code Targeting

Our results indicate that researchers can successfully recruit diverse individuals for web-based health-related studies using Facebook advertising campaigns with zip code targeting. By placing advertisements in zip codes ranked first on the percentage of households of the target ethnicity and then on the focal health behavior (in this case, cigarette expenditure per household), we successfully increased our outreach to and yield of black/African American– and Hispanic/Latino applicants. Table 1 shows our expected yields if we had continued these campaigns for 2 years, spending US $20 per day or US $140 per week, compared with our baseline general market campaign. Although we were concerned about higher costs, costs were not significantly higher for these ethnic-focused campaigns compared with our baseline general market campaign. We also recruited Hispanic/Latinos using Facebook’s option to reach Spanish language speakers, and this produced a similar yield to our Hispanic/Latino campaign using zip codes. But it was more costly to produce this yield, perhaps because our study required English fluency.

**Table 1:** Estimated campaign yields based on spending US $20/day on each campaign for 2 years.

Yield type and campaign^a^		White, n (%)	Black/African American, n (%)	Hispanic/Latino, n (%)	Total^b^, N
**Queries**					
	General market	2005 (84.5)	294 (12.4)	74 (3.1)	2373
	Black/African American	1125 (51.9)	986 (45.5)	58 (2.7)	2169
	Hispanic/Latino	1258 (66.9)	301 (16.0)	322 (17.1)	1881
	Spanish language	730 (62.0)	62 (5.3)	385 (32.7)	1177
**Applicants**					
	General market	428 (86.6)	56 (11.3)	10 (2.0)	494
	Black/African American	269 (68.1)	115 (29.1)	11 (2.8)	395
	Hispanic/Latino	249 (77.3)	36 (11.2)	37 (11.5)	322
	Spanish language	185 (72.5)	16 (6.3)	54 (21.2)	255
**Participants**					
	General market	83 (87)	9 (9)	3 (3)	95
	Black/African American	28 (70)	11 (27)	1 (2)	40
	Hispanic/Latino	22 (81)	4 (14)	1 (3)	27
	Spanish language	29 (87)	0 (0)	4 (12)	33

^a^The estimates are based on the yield of US $140/week (US $20/day) multiplied by 104 weeks to show estimated results for 2 years. The estimates in this table come from the observed yields of these campaigns extrapolated to reflect a standard expenditure of US $20/day for 2 years. The observed yields are based on US $77,133 spent, out of the US $87,425 in total spending on Facebook for the randomized controlled trial. The remainder was spent before the Facebook Pixel install or on weeks or test campaigns not reported here. The US $77,133 spent was divided up across campaigns as follows: general market 427 days at US $126/day, black/African American 427 days at US $23/day, Hispanic/Latino 427 days at US $23/day, and Spanish language 105 days at US $36/day.

^b^Row percentages add up to 100. Other ethnicities that were recruited during the campaigns were not factored into these estimates.

### Principal Findings on Audience Network Placement and Scandal Effects

This study also found that Facebook’s recommended audience network placement, although cheaper at producing advertisement clicks, was far more expensive at producing website queries. This means that many individuals who clicked on our advertisements were not interested. Many individuals may have clicked on our advertisements to use a free app (eg, play a game), not because they were interested in our study. Thus, we do not recommend using the audience network placement. We also learned that the Facebook privacy scandal involving Cambridge Analytica had a negative impact on recruitment for our campaigns that were black/African American–focused and Hispanic-/Latino-focused. This scandal increased our cost per query and cost per applicant. Hence, researchers should keep Facebook scandals in mind, as they may raise recruitment costs.

### Principal Findings on Audience Lookalike Feature

Facebook’s recommended audience lookalike feature produced advertisement clicks at a lower price point than our baseline general market campaign; however, query and applicant price points were similar to our general market campaign. Researchers may want to consider using Facebook’s audience lookalike feature because it may cost less at the advertisement click stage, if this is a high priority, and if the required *source audience* can be identified based on website behavior as tracked by a Facebook Pixel (we used queries on our website). Potential drawbacks to this feature include privacy and institutional review board (IRB) concerns. Facebook’s audience lookalike feature seems to involve using data collected whenever people are logged into Facebook, including data that are both public and relatively more private, including likes, posts, visits to other websites, friend networks, and friends’ web-based behavior. This data use could raise potential privacy and IRB approval issues because it is not possible in IRB consent forms or information sheets to describe to potential participants what Facebook data of theirs will be used for the audience lookalike feature (this is proprietary) and it is not possible to obtain consent from their Facebook friend networks.

### Principal Findings on Advertising Costs

Facebook-based recruitment for health-related research has been the focus of previous studies, allowing for cost and yield comparisons with our study. Looking at cost per advertisement click, Whitaker et al [21] reported a mean of US $0.57 across several studies (range US $0.20-US $1.74), whereas Ramo and Prochaska reported a mean of US $0.45 [35]. Our costs per advertisement click across campaigns are within this range (US $0.67, SD 0.19-US $1.56, SD 0.34). Cost per participant varies widely; Whitaker et al [21] found that the cost per participant ranged from US $1.36 to US $110.00 depending on study length and engagement. Although our cost per participant ranged from US $121.05 (SD 60.02) to US $171.66 (SD 97.37), we recruited for a 3-month study that involved daily engagement in a web-based group and complete cessation from smoking. We were able to generate detailed cost and yield findings because we directed our website developer to install a Facebook Pixel on our study website. This Pixel allowed us to track which Facebook advertisement campaign a query came from and determine whether the individual who queried applied to the study and participated. We, therefore, recommend that researchers install the Facebook Pixel.

### Study Limitations and Strengths

Our findings on recruitment yields standardize for spending at US $140 per week, so they are relatively independent of costs. However, our findings on recruitment costs are limited by the fact that recruitment costs are strongly affected by study inclusion/exclusion criteria, participation time demands, and study benefits and incentives. Recruitment costs can also fluctuate daily and seasonally. In addition, we recruited throughout the continental United States for our web-based study, and studies that are more local may yield different outcomes. Moreover, our costs per yield for a Spanish language campaign may be overestimated because English fluency was a requirement for inclusion in our study. Studies with translated materials and no English-speaking requirements may find higher enrollments and lower costs.

A strength of our study is that it was a national study with a large sample size; 980 people were recruited overall. Furthermore, we examined Facebook advertising campaigns considering both yields and costs and considered 4 separate outcomes: advertisement clicks, queries, applicants, and participants. In addition, our study lasted for 61 weeks, allowing us to compare costs of Facebook recruitment both before and after a major Facebook privacy scandal. Our main goal was to innovatively test the efficacy of targeting specific zip codes on Facebook to reach ethnically or racially diverse populations. Advertiser intent can have ethical ramifications when utilizing zip code targeting, especially when protected characteristics are targeted such as race or ethnicity. As of August 2019, Facebook restricts audience targeting options, including zip code targeting in the case of housing, credit, or employment to help prevent discrimination [36]. In our case, advertiser intent is heath promoting, and therefore zip code targeting is justifiable.

### Conclusions

Our main conclusion is that Facebook advertising campaigns that employ suitable zip code targeting can help to find and recruit blacks/African Americans and Hispanics/Latinos for web-based health-related studies.

## Appendix

Multimedia Appendix 1 Facebook advertising campaign timeline.

Multimedia Appendix 2 CONSORT diagrams for participants by Facebook campaign.

## Abbreviations

IRB: institutional review board
