# Supplementary material for: Facebook Recruitment Using Zip Codes to Improve Diversity in Health Research: Longitudinal Observational Study
Source: J Med Internet Res. 2020 Jun 5;22(6):e17554. doi: 10.2196/17554 (PMC7305557; doi:10.2196/17554)
Supplement: Multimedia Appendix 1 [file jmir_v22i6e17554_app1.docx]

Multimedia Appendix 1

Facebook advertising campaign timeline.

Note: Gray cells indicate a running campaign during that week. We did not run the campaigns during the Thanksgiving and Christmas

holidays. *The Cambridge Analytica scandal occurred in 3/18. Some weeks were excluded from cost analyses due to no responses

received that week. We did not include these weeks as 0’s would artificially lower our cost estimates.

C = Advertisement clicks, Q = Queries, A = Applicants, P = Participants.
