# Supplementary material for: Facebook Recruitment Using Zip Codes to Improve Diversity in Health Research: Longitudinal Observational Study
Source: J Med Internet Res. 2020 Jun 5;22(6):e17554. doi: 10.2196/17554 (PMC7305557; doi:10.2196/17554)
Supplement: Multimedia Appendix 2 [file jmir_v22i6e17554_app2.docx]

**Figure 1. Consort for general market campaign participants**

**Multimedia Appendix 2**

Queried n=9,500 (14.2%)

 Filled out interest form

Race/Ethnicity:

- White n=7,349 (77.3%)
- Black/African American n=1,081 (11.4%)
- Hispanic/Latino n=271 (2.9%)
- Other n=799 (8.4%)

Saw ad (n=2,867,447)

Clicked ad (n=66,681) (2.3%)

Non-Applicants n=7,628 (80.3%)

 Declined to take screening survey

Race/Ethnicity:

- White n=5,808 (76.1%)
- Black/African American n=878 (11.5%)
- Hispanic/Latino n=235 (3.1%)
- Other n=707 (9.3%)

Participants n=389 (20.8%)

 Completed screening survey and phone and email verifications

Race/Ethnicity:

- White n=328 (84.3%)
- Black/African American n=35 (9.0%)
- Hispanic/Latino n=11 (2.8%)
- Other n=15 (3.9%)

Applicants n=1,872 (19.7%)

 Took screening survey

Race/Ethnicity:

- White n=1,541 (82.4%)
- Black/African American n=203 (10.8%)
- Hispanic/Latino n=36 (1.9%)
- Other n=92 (4.9%)

Queried n=57,181 (85.8%)

 Did not fill out interest form

Race/Ethnicity: unknown

Non-Participants n=1,483 (79.2%)

Reasons for failing to enroll*:

- Didn’t Finish Screening Survey (n=598) (40.3%)
- Didn’t Consent (n=289) (19.5%)
- Failed/Didn’t do Webcam/Phone/Email Verification (n=264) (17.8%)
- Health Contraindicated (n=293) (17.8%)
- Daily Marijuana or Any Hard Drug Use (n=71) (4.8%)
- On Smoking Cessation Drugs or NRT (n=40) (2.7%)
- No Access to Internet (n=34) (2.3%)
- Less than 5 Cigs/Day (n=28) (1.9%)
- Other (n=53) (3.6%)

Race/Ethnicity:

- White n=1,213 (81.8%)
- Black/African American n=168 (11.3%)
- Hispanic/Latino n=25 (1.7%)
- Other n=77 (5.2%)

*Note: Applicants could fail on multiple questions

**Figure 2. Consort for black/African American campaign participants**

Queried n=1,617 (12.9%)

 Filled out interest form

Race/Ethnicity:

- White n=750 (46.4%)
- Black/African American n=657 (40.6%)
- Hispanic/Latino n=39 (2.4%)
- Other n=171 (10.6%)

Queried n= 10,927 (87.1%)

 Did not fill out interest form

Race/Ethnicity: unknown

Saw ad (n=222,508)

Clicked ad (n=12,544) (5.6%)

Non-Applicants n=1,319 (81.6%)

 Declined to take screening survey

Race/Ethnicity:

- White n=558 (42.3%)
- Black/African American n=575 (43.6%)
- Hispanic/Latino n=31 (2.3%)
- Other n=155 (11.8%)

Participants n=58 (19.5%)

 Completed screening survey and phone and email verifications

Race/Ethnicity:

- White n=39 (67.2%)
- Black/African American n=15 (25.9%)
- Hispanic/Latino n=1 (1.7%)
- Other n=3 (5.2%)

Applicants n=298 (18.4%)

 Took screening survey

Race/Ethnicity:

- White n=192 (64.4%)
- Black/African American n=82 (27.5%)
- Hispanic/Latino n=8 (2.7%)
- Other n=16 (5.4%)

Non-Participants n=240 (80.5%)

Reasons for failing to enroll*:

- Didn’t Finish Screening Survey (n=87) (36.3%)
- Failed/Didn’t do Webcam/Phone/Email Verification (n=59) (24.6%)
- Health Contraindicated (n=43) (17.9%)
- Didn’t Consent (n=37) (15.4%)
- Daily Marijuana or Any Hard Drug Use (n=17) (7.1%)
- On Smoking Cessation Drugs or NRT (n=8) (3.3%)
- Less than 5 Cigs/Day (n=6) (2.5%)
- No Access to Internet (n=3) (1.3%)
- Other (n=9) (3.8%)

Race/Ethnicity:

- White n=153 (63.8%)
- Black/African American n=67 (27.9%)
- Hispanic/Latino n=7 (2.9%)
- Other n=13 (5.4%)

*Note: Applicants could fail on multiple questions

**Figure 3. Consort for Hispanic/Latino campaign participants**

Queried n=1,422 (13.0%)

 Filled out interest form

Race/Ethnicity:

- White n=840 (59.1%)
- Black/African American n=200 (14.1%)
- Hispanic/Latino n=215 (15.1%)
- Other n=167 (11.7%)

Queried n= 9,547 (87.0%)

 Did not fill out interest form

Race/Ethnicity: unknown

Saw ad (n=383,713)

Clicked ad (n=10,969) (2.9%)

Non-Applicants n=1,172 (82.4%)

 Declined to take screening survey

Race/Ethnicity:

- White n=663 (56.7%)
- Black/African American n=174 (14.8%)
- Hispanic/Latino n=188 (16.0%)
- Other n=147 (12.5%)

Participants n=43 (17.2%)

 Completed screening survey and phone and email verifications

Race/Ethnicity:

- White n=33 (76.7%)
- Black/African American n=6 (14.0%)
- Hispanic/Latino n=1 (2.3%)
- Other n=3 (7.0%)

Applicants n=250 (17.6%)

 Took screening survey

Race/Ethnicity:

- White n=177 (70.8%)
- Black/African American n=26 (10.4%)
- Hispanic/Latino n=27 (10.8%)
- Other n=20 (8.0%)

Non-Participants n=207 (82.8%)

Reasons for failing to enroll*:

- Didn’t Finish Screening Survey (n=74) (35.7%)
- Didn’t Consent (n=41) (19.8%)
- Health Contraindicated (n=39) (18.8%)
- Failed/Didn’t do Webcam/Phone/Email Verification (n=33) (15.9%)
- Daily Marijuana or Any Hard Drug Use (n=11) (5.3%)
- On Smoking Cessation Drugs or NRT (n=8) (3.9%)
- Less than 5 Cigs/Day (n=8) (3.9%)
- No Access to Internet (n=7) (3.4%)
- Other (n=9) (4.3%)

Race/Ethnicity:

- White n=144 (69.5%)
- Black/African American n=20 (9.7%)
- Hispanic/Latino n=26 (12.6%)
- Other n=17 (8.2%)

*Note: Applicants could fail on multiple questions

**Figure 4. Consort for Spanish language campaign participants**

Queried n=359 (14.5%)

 Filled out interest form

Race/Ethnicity:

- White n=188 (52.3%)
- Black/African American n=16 (4.5%)
- Hispanic/Latino n=99 (27.6%)
- Other n=56 (15.6%)

Queried n= 2,124 (85.5%)

 Did not fill out interest form

Race/Ethnicity: unknown

Saw ad (n=89,128)

Clicked ad (n=2,483) (2.8%)

Non-Applicants n=289 (80.5%)

 Declined to take screening survey

Race/Ethnicity:

- White n=140 (48.4%)
- Black/African American n=12 (4.2%)
- Hispanic/Latino n=85 (29.4%)
- Other n=52 (18.0%)

Participants n=16 (22.9%)

 Completed screening survey and phone and email verifications

Race/Ethnicity:

- White n=14 (87.5%)
- Black/African American n=0 (0%)
- Hispanic/Latino n=2 (12.5%)
- Other n=0 (0%)

Applicants n=70 (19.5%)

 Took screening survey

Race/Ethnicity:

- White n=48 (68.6%)
- Black/African American n=4 (5.7%)
- Hispanic/Latino n=14 (20.0%)
- Other n=4 (5.7%)

Non-Participants n=54 (77.1%)

Reasons for failing to enroll*:

- Health Contraindicated (n=20) (37.0%)
- Didn’t Finish Screening Survey (n=15) (27.8%)
- Failed/Didn’t do Webcam/Phone/Email Verification (n=12) (22.2%)
- Didn’t Consent (n=9) (16.7%)
- Daily Marijuana or Any Hard Drug Use (n=4) (7.4%)
- On Smoking Cessation Drugs or NRT (n=2) (3.7%)
- Less than 5 Cigs/Day (n=2) (3.7%)
- No Access to Internet (n=1) (1.9%)
- Other (n=2) (3.7%)

Race/Ethnicity:

- White n=34 (63.0%)
- Black/African American n=4 (7.4%)
- Hispanic/Latino n=12 (22.2%)
- Other n=4 (7.4%)

*Note: Applicants could fail on multiple questions
